# Bladder neck contracture: incidence, mechanisms, and therapeutic advances

**DOI:** 10.1097/MS9.0000000000003930

**Published:** 2025-09-23

**Authors:** Yize Ren, Menghua Wu, Liancheng Jia, Hongliang Rui

**Affiliations:** a Beijing Hospital of Traditional Chinese Medicine, Capital Medical University, Beijing, China; b Beijing Institute of Chinese Medicine, Beijing, China; c Department of Urology, Beijing Hospital of Traditional Chinese Medicine, Capital Medical University, Beijing, China

**Keywords:** bladder neck contracture, fibrosis mechanisms, minimally invasive endoscopy, regenerative medicine, robot-assisted surgery

## Abstract

Bladder neck contracture (BNC) is a progressive narrowing of the bladder neck and adjacent posterior urethra, primarily caused by fibrotic tissue proliferation and scar formation. It commonly occurs following prostate surgery or radiotherapy. Its pathogenesis involves acute and chronic inflammatory responses triggered by surgical or radiation-induced injury, transdifferentiation of fibroblasts into myofibroblasts, and excessive extracellular matrix (ECM) deposition, mediated by the interplay of transforming growth factor beta (TGF-β)/Smad signaling and mechanotransduction feedback. Clinically, patients present with voiding difficulty, a weakened urinary stream, increased post-void residual volume, and urinary retention, which may be complicated by urinary tract infections, bladder stones, and renal impairment. Diagnosis is based on medical history, urodynamic studies, ultrasound, voiding cystourethrography(VCUG), and cystoscopic evaluation. Treatment follows a stepwise approach ranging from conservative management to endoscopic and then surgical interventions. First-line therapies include transurethral dilation or incision, often combined with local anti-scar agents and intermittent self-catheterization. Refractory or recurrent cases may require open or robot-assisted reconstructive surgery. Robot-assisted surgery, with its high-definition 3D visualization, multi-degree-of-freedom instrumentation, and precise suturing, significantly reduces the incidence of BNC and provides a minimally invasive option for complex reconstructions. Future research directions focus on regenerative medicine, tissue-engineered scaffolds, and anti-fibrotic pharmacotherapy, aiming to fundamentally interrupt the fibrotic cascade. Personalized risk stratification and early intervention are expected to enhance long-term outcomes and quality of life.

Bladder neck contracture (BNC) is a form of urethral stricture resulting from fibrotic tissue proliferation and scar formation in the bladder neck and posterior urethra^[1]^. It commonly occurs following surgical interventions for benign prostatic hyperplasia (BPH) and prostate cancer^[2]^, and is also a frequent complication of urethral and pelvic surgeries. BNC can lead to severe lower urinary tract symptoms, urinary retention, upper urinary tract deterioration, and even renal failure. In recent years, advances in imaging, urodynamics, and endoscopic technologies have improved diagnostic and therapeutic accuracy. Molecular studies have identified the transdifferentiation of fibroblasts into myofibroblasts as a critical event in scar contracture. Minimally invasive surgical techniques, particularly robot-assisted laparoscopic procedures, have had a significant impact on the prevention and treatment of BNC by enhancing anastomotic quality through high-definition visualization and precise manipulation, while minimizing tissue trauma and recurrence risk. These technological advantages also contribute to a reduced risk of postoperative complications^[3]^. We conducted a comprehensive review by searching PubMed, Embase, and the Cochrane Library for the terms “bladder neck contracture,” “vesicourethral anastomotic stenosis,” “bladder neck stenosis,” and “prostatectomy complication,” screening articles published between 2000 and 2024 and updating the search through June 2025. We also delineate our inclusion and exclusion criteria: we included original clinical studies (prospective and retrospective), systematic reviews/meta-analyses, and relevant guidelines published in English, while excluding single-case reports and non-English literature to maintain a focus on high-impact evidence. This review summarizes the etiologies of BNC, the surgical techniques influencing its development, and the role of robotic surgery, with the aim of improving patient outcomes. In accordance with the TITAN Guidelines 2025, this work complies with the governing declaration and ethical use of AI^[4]^.

**HIGHLIGHTS**

- Bladder neck contracture is a fibrotic narrowing of the bladder neck and posterior urethra after surgery or radiotherapy, with incidence dropping from 0.3% to 12.3% after transurethral resection of the prostate to under 1% in robot-assisted procedures.
- It arises from acute and chronic inflammation, fibroblast-to-myofibroblast differentiation, excessive collagen deposition, and activation of TGF-β/Smad and mechanotransduction pathways.
- Diagnosis uses ultrasound, voiding cystourethrography, and cystoscopy, and treatment escalates from α-blockers and bladder training to endoscopic dilation/incision with antifibrotic agents, then to open or robot-assisted reconstruction.
- Future work will merge regenerative medicine, tissue-engineered scaffolds, and antifibrotic drugs to halt fibrosis and improve long-term patency and quality of life.

## Etiology and epidemiology

Bladder neck contracture(BNC) is a complex fibrotic disorder arising from the interaction of multiple intrinsic and extrinsic factors, with iatrogenic causes accounting for the vast majority of cases. Clinically, over 95% of BNC cases are attributable to abnormal wound healing following prostate or pelvic surgeries and radiotherapy. The common underlying pathology involves local tissue injury induced by surgical or radiation trauma, which triggers acute inflammation, excessive fibroblast activation, transdifferentiation into myofibroblasts, and abundant extracellular matrix(ECM) deposition, ultimately leading to irreversible fibrosis and luminal narrowing.

Transurethral resection of the prostate (TURP), previously the gold standard for benign prostatic hyperplasia(BPH), has been closely associated with BNC, with reported postoperative incidence ranging from 0.3% to 12.3%^[5,6]^. Mechanistically, thermal injury during resection, improper tissue handling, postoperative inflammation, and urinary extravasation all contribute to BNC development. In recent years, newer surgical technologies such as holmium laser enucleation (HoLEP), thulium laser enucleation (ThuLEP), photoselective vaporization (PVP), and bipolar resection have significantly reduced BNC incidence by enabling precise tissue removal with minimal thermal spread and reduced intraoperative bleeding. Some studies report BNC rates as low as 3.3%–4.1%^[7–9]^, though surgeon experience, anatomical variation, and postoperative care remain important influencing factors.

Vesicourethral anastomotic stricture (VUAS) following radical prostatectomy for prostate cancer is a distinct subtype of BNC. Its incidence after open radical prostatectomy (ORP) ranges from 2.5% to 8.4%, depending largely on surgical technique and experience^[10,11]^. While laparoscopic radical prostatectomy(LRP) offers improved visualization and precision, it has not consistently lowered VUAS rates compared to ORP^[12]^. In contrast, robot-assisted radical prostatectomy (RARP) represents a major milestone in reducing VUAS incidence, with large-scale studies reporting rates below 1%, and in some high-volume centers, approaching zero^[5,13]^. Nevertheless, anastomotic techniques, intraoperative blood loss, operative time, surgeon proficiency, and patient-specific factors continue to affect outcomes.

Pelvic radiotherapy, whether used as definitive treatment for prostate cancer or as adjuvant therapy for other malignancies, can also lead to BNC. Radiation-induced BNC is often insidious, with symptoms emerging for months or even years of post-treatment. Its pathogenesis involves microvascular damage to the bladder neck and surrounding tissues, resulting in ischemia, chronic inflammation, and fibrosis^[5]^. The incidence of radiation-associated BNC correlates with total dose, fractionation schedule, radiation field, and concurrent use of hormonal or chemotherapeutic agents. These cases tend to present with more extensive and denser fibrosis, lower responsiveness to endoscopic therapy, higher recurrence rates, and increased treatment complexity^[14–16]^.

Although rare, non-iatrogenic causes of BNC include chronic prostatitis, posterior urethritis, and recurrent urinary tract infections, which may induce local edema, hyperemia, and chronic inflammation^[1]^. These stimuli can activate fibroblasts to transform into myofibroblasts and secrete ECM, resulting in progressive fibrosis^[17]^. Traumatic injuries—such as pelvic fractures, straddle injuries, or perineal contusions—may cause direct damage to the bladder neck or posterior urethra, and subsequent abnormal wound healing can result in excessive scarring and stricture formation. Congenital BNC is extremely uncommon and typically presents during childhood or adolescence with voiding difficulties^[18]^. Idiopathic BNC refers to cases with no identifiable etiology after thorough exclusion of known causes; these may involve genetic predispositions or autoimmune processes that warrant further investigation.

The incidence and prevalence of BNC vary widely due to differences in diagnostic criteria, surgical techniques, and follow-up durations. Before the robotic surgery era, BNC was relatively common after ORP, and TURP-associated BNC rates reached as high as 12.3%. With the emergence of bipolar vaporization and other refined techniques, the incidence has dropped to 3.3%–4.1%. Robotic surgery, particularly in high-volume centers, has further reduced BNC rates—sometimes to near zero. These trends underscore the role of surgical innovation in mitigating BNC risk. However, radiation-associated BNC may be underreported due to its delayed presentation and inadequate follow-up. In prostate cancer patients receiving salvage therapies, BNC remains a significant concern, with reported incidence ranging from 7% to 47% (Table 1).

**Table 1:** Influence of different inducements on bladder neck contracture

Inducement/operation	Incidence of BNC (range)	References
After traditional TURP	0.3%–12.3%	^[5,6]^
After laser/plasma surgery	3.3%–4.1%	^[7–9,19]^
VUAS occurs after ORP	2.5%–8.4%	^[10,11,20–27]^
VUAS occurs after RARP	<1%	^[5,13]^
Radiotherapy related	1.7%–5.2%; it can reach 7%–47% in patients with salvage treatment	^[28–31]^

The data of open surgery are taken from the literature before the rise of robotic surgery, and the high value can only be found in high-risk situations such as radiotherapy; modern BPH surgery includes plasma bipolar electrotomy, laser vaporization, and laser enucleation.

## Risk factors

Understanding why incidence varies so markedly leads naturally to a discussion of modifiable and non-modifiable risk factors. The potential risk factors for bladder neck contracture(BNC) can be broadly categorized into patient-related and surgery-related factors. Diabetes significantly increases BNC risk via microvascular damage, hyperglycemia-induced collagen dysregulation, and impaired immune function^[32]^. Coronary artery disease and hypertension further compound these effects by impairing anastomotic blood flow and promoting fibrosis^[33]^. Lifestyle factors such as smoking—one of the strongest independent predictors—contribute through tissue hypoxia and delayed wound healing^[32,34–36]^. Obesity (BMI ≥ 30) and advanced age (> 70 years) are also associated with elevated risk due to systemic inflammation, insulin resistance, and diminished regenerative capacity^[28,35,37]^.

Preoperative urinary tract infections or prostatitis, as well as a history of pelvic surgery or radiotherapy, increase the risk of BNC via chronic inflammation and tissue ischemia^[1]^. For reoperations, BNC risk may rise to 22%–40%^[15,38]^. Notably, prostate size also affects risk, with smaller prostates (< 30 g) predisposing to BNC after transurethral surgery^[1,39]^.

Surgical technique remains the most critical determinant. Open procedures carry a higher BNC incidence (2.6%–7.5%) compared to robot-assisted surgery (0%–2.1%)^[40,41]^. Tension-free mucosal anastomosis and precise alignment are key^[3]^; even minor misalignments > 1 mm triple the risk^[42]^. Inappropriate use of electrocautery, extended operation times, excessive blood loss, and perioperative complications like leakage or hematoma formation further elevate BNC likelihood^[2,43–45]^. Additional contributors include prolonged catheterization, low irrigation temperatures, and migration, especially Hem-o-lok clips in robotic surgery^[22]^.

A comprehensive understanding of these risk factors enables individualized preoperative risk assessment and targeted perioperative strategies to prevent BNC. Radiation-associated BNC poses a unique challenge due to its delayed onset and the progressive nature of radiation-induced fibrosis, which may persist for 5–10 years^[46]^. In salvage settings after curative radiotherapy, BNC risk can reach 20%–30%^[14–16]^. Table 2 presents odds ratios/absolute risks for each factor, enabling rapid comparison.

**Table 2:** Risk factors of BNC

Patient-related factors
Age > 70 yr—doubles risk versus < 60 yr.
Current smoking—OR ≈ 3.5; prevalence 26% versus 8% in nonsmokers.
Diabetes mellitus—microvascular compromise impairs healing.
Coronary artery disease/hypertension—additive vascular burden.
Obesity (BMI ≥ 30)—chronic systemic inflammation and technical difficulty.
Procedure-related factors
Small prostate (<30 g) at TURP—higher residual thermal injury.
Excessive electrocautery or prolonged operative time—greater tissue devitalization.
Intra-operative blood loss > 500 mL—surrogate for difficult dissection.
Re-catheterization or urinary leak—OR ≈ 5.6 for subsequent BNC.
Salvage surgery after radiotherapy or HIFU—ischemia-fibrosis cycle.

## Pathophysiological changes and mechanisms

### Etiology and risk factors

Bladder neck contracture(BNC) arises from a complex cascade involving multifactorial tissue injury, dysregulated inflammation, aberrant wound healing, and pathological fibrosis^[17]^. Acute insults—mechanical trauma, thermal or radiation injury, and ischemia-reperfusion—provoke a local inflammatory storm^[47]^. If unresolved, this response becomes chronic, activating fibroblasts into ECM-secreting myofibroblasts that generate dense, rigid scar tissue replacing the normal bladder neck architecture^[48–51]^.

Multiple sources of injury—including surgical trauma^[52,53]^, thermal spread^[1,34]^, oxidative stress^[17,54]^, radiation^[17,55]^, and urinary extravasation^[56]^—trigger the release of damage associated molecular patterns (DAMPs)^[57]^and activation of TLR/NLR signaling, leading to nuclear factor kappa-B (NF-κB)-mediated cytokine release and immune cell recruitment^[49,58–61]^. This inflammatory surge, if persistent, sets the stage for sustained fibrosis^[62]^.

### Fibrogenic pathways

TGF-β1/Smad signaling governs fibroblast-to-myofibroblast differentiation and matrix production^[44,63–66]^. This is amplified by other pro-fibrotic pathways [Wnt/β-catenin, platelet-derived growth factor (PDGF), connective tissue growth factor (CTGF), and Hippo-yes-associated protein (YAP)/transcriptional coactivator with PDZ-binding motif (TAZ)], mechanical feedback, and hypoxia-induced Hypoxia-Inducible Factor (HIF) signaling^[67–69]^. Collagen crosslinking and ECM accumulation are compounded by matrix metalloproteinase (MMP)/tissue inhibitor of metalloproteinase (TIMP) imbalance, further enhancing tissue stiffness^[70,71]^.

**Figure 1.**
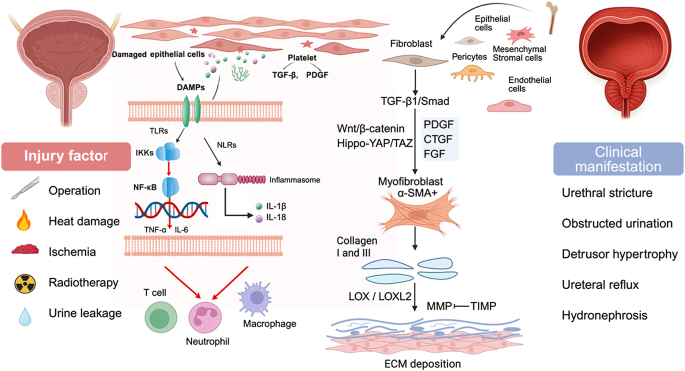
Pathophysiological progression of bladder neck fibrosis. Stage 1: Injury factor as indicated triggers acute inflammation. Damaged cells release DAMPs, activating TLRs/NLRs and NF-κB signaling, inducing TNF-α, IL-1β, and IL-6. Coagulation activates platelets to release TGF-β and PDGF. Infiltrating neutrophils, M2 macrophages, and T cells sustain chronic low-grade inflammation and initiate fibrogenesis. Stage 2: Chronic inflammation induces fibroblast activation into α-SMA^+^ myofibroblasts, promoting ECM synthesis and contraction. TGF-β1/Smad is central, with PDGF, CTGF, FGF, Wnt/β-Catenin, and YAP/TAZ pathways reinforcing the process. TGF-β activation of its receptor leads to SMAD-mediated transcription of collagen and other extracellular matrix components, promoting scar formation. EMT, EndMT, and bone marrow-derived fibroblasts contribute. Hypoxia, tension, and epigenetic changes form a self-amplifying loop. Stage 3: Myofibroblasts secrete excess collagen I/III, causing disorganized ECM and progressive cross-linking. MMP activity is inhibited by TIMPs, while LOX and LOXL2 increase stiffness. Fibrotic remodeling narrows the lumen, impairs voiding, and leads to BNC. DAMPs, damage associated molecular patterns; EMT, epithelial-mesenchymal transition; EndMT, endothelial-mesenchymal transition; TGF-β, transforming growth factor beta; ECM, extracellular matrix; MMPs, matrix metalloproteinases; TLRs, toll-like receptors; NLRs, NOD-like receptors; IKKs, IκB kinase; NF-κB, nuclear factor kappa-B; TNF-α, tumor necrosis factor-alpha; IL-1β, interleukin-1 beta; IL-6, interleukin-6; IL-18, interleukin-18; PDGF, platelet-derived growth factor; CTGF, connective tissue growth factor; FGF, fibroblast growth factor; YAP, Yes-associated protein; TAZ, transcriptional coactivator with PDZ-binding motif; TIMPs, tissue inhibitor of metalloproteinase; α-SMA^+^, α-Smooth muscle actin^+^; LOX, lysyloxidase; LOXL2, lysyloxidase 2.

Structurally, progressive fibrosis narrows the bladder neck, causing bladder outlet obstruction (BOO). Compensatory detrusor hypertrophy gives way to muscle fatigue and fibrosis, reducing compliance and bladder function^[72,73]^. Secondary effects include upper tract deterioration from backpressure and infection, culminating in renal failure^[74,75]^. These sequential events, from molecular dysregulation to organ and system-level complications, present opportunities for targeted intervention that could fundamentally reshape BNC outcomes (Fig. 1).

## Clinical manifestations and diagnosis

The clinical presentation of bladder neck contracture(BNC) is largely attributable to lower urinary tract obstruction and its complications^[76]^. Symptoms typically emerge several weeks to months following prostatectomy, with voiding difficulty as the predominant complaint^[21,77,78]^. Patients often report delayed initiation of micturition, straining with abdominal pressure, weak or narrow urinary stream, reduced trajectory, interrupted flow, and intermittent dribbling—all suggestive of mechanical BOO^[79]^. Increased post-void residual (PVR) volume is a key marker and can be identified on ultrasound as incomplete bladder emptying. In more advanced cases, urinary retention may develop, characterized by suprapubic distension, pain, and inability to void; some patients initially present with acute urinary retention^[45,80,81]^.

Recurrent urinary tract infections are common due to stagnant urine, often presenting with irritative symptoms such as urgency, frequency, and dysuria^[43]^. Chronic obstruction can also lead to morphological changes including bladder trabeculation, diverticula, and stone formation, with calculi aggravating hematuria and voiding dysfunction^[82]^. Overflow incontinence may occur in patients with BNC and must be differentiated from true stress urinary incontinence (SUI): the latter typically results from sphincteric damage in the early postoperative period, while the former is associated with impaired flow and may transiently worsen after obstruction relief^[83–86]^.

A multimodal diagnostic approach is required. Urodynamic testing reveals reduced maximum flow rate (Qmax) and an obstructive pattern^[87]^. Ultrasound helps assess residual urine volume and potential hydronephrosis^[88]^. Voiding cystourethrography (VCUG) can visualize a constrictive ring at the bladder neck and obstructed contrast flow during voiding^[89]^. Cystoscopy remains the gold standard for confirmation, allowing direct assessment of scar morphology and severity—often appearing as a pinpoint narrowing in advanced cases^[90,91]^. In practice, uroflowmetry and ultrasound serve as initial screening tools, followed by cystoscopy for definitive diagnosis. VCUG may be added for surgical planning in complex cases^[90,92]^. It is important to note that within three months postoperatively, inflammatory reactions may obscure clinical judgment, requiring dynamic monitoring and reassessment^[90]^. Therefore, a “second-look” cystoscopy at 6–8 weeks after endoscopic incision allows early detection of re-narrowing when lumen diameter falls below 14 Fr, enabling prompt repeat incision before dense fibrosis recurs.

## Treatment

### Minimally invasive management

The management of bladder neck contracture(BNC) should be individualized based on stricture severity, symptom burden, and treatment history. The overarching goals are to restore unobstructed bladder outlet function and prevent complications^[17]^. Treatment generally follows a stepwise approach from conservative to surgical interventions^[2]^.

Conservative management is suitable for patients with mild symptoms, limited narrowing, and preserved voiding function^[93]^. It includes α1-adrenergic blockers (e.g., tamsulosin, terazosin) to relax bladder neck smooth muscle^[94,95]^, antibiotics for concurrent urinary tract infections^[96]^, and cautious use of anticholinergics to control detrusor overactivity^[97]^. Yang *et al.* reported that 88 elderly patients with refractory urinary tract infections who received treatment with tamsulosin combined with levofloxacin showed a total effective rate of 59%–67% after a 9-month follow-up^[98]^. Bladder training—comprising scheduled voiding, nocturnal fluid restriction, and double voiding—can reduce PVR urine. Pelvic floor muscle training may prevent postoperative incontinence but has no direct effect on the stricture itself^[99]^. For patients with minimal PVR and preserved renal function, a watchful waiting strategy may be acceptable, provided that ultrasound and urodynamic surveillance are regularly performed^[100]^. However, conservative therapy does not reverse fibrotic narrowing, and progression warrants timely surgical intervention^[76]^.

Minimally invasive surgery is the cornerstone of treatment for moderate to severe BNC or failure of conservative therapy. Initial management of short, non-obliterative BNC frequently starts with sound or high-pressure balloon dilation (24–30 Fr). Success rates range 55%–84% at 6- to 12-month follow-up^[101]^, but cumulative recurrence approaches 70% by 24 months^[102]^. Intermittent self-catheterization is often recommended postoperatively but suffers from poor patient adherence. Park *et al.* treated 32 post-prostatectomy cases: single-session patency 93% at 3 months, falling to 62% at 18 months; repeat dilation restored patency in another 22%. Cao *et al.* reported that among 53 patients with BNC who underwent endoscopic incision Transurethral incision of bladder neck (TUIBN) with cold knife or electrocautery to release scar tissue after surgery, 5 patients had a recurrence of BNC within 6 months of follow-up, and 5 out of 30 patients had a recurrence of BNC after 1 year of follow-up. The recurrence rate was relatively high, approximately 9.4% to 16.7%^[103]^. Ramirez *et al.* reported that among 50 patients with BNC, the modified techniques such as balloon dilation combined with symmetrical incision enabled 72% of the patients to achieve success in one operation^[36]^. Cold-knife or electrocautery incision at 3 and 9 o’clock remains standard for moderate strictures. Single-procedure success ≈ 65%–70%; repeat incision lifts cumulative success to 85%. Deep lateral cold-knife incision to perivesical fat^[36]^( *n* = 75) achieved 82% long-term patency with < 4% *de-novo* SUI. Laser technologies (holmium and thulium) provide precise dissection and hemostasis, though recurrence remains a challenge^[104]^. Holmium or thulium laser provides precise tissue ablation and hemostasis. A multicenter series (48 pts) reported 86% patency (median 14 months), with 2.9% single-procedure recurrence. Vanni *et al.* reported that among 18 patients who underwent bladder neck incision and mitomycin C injection, with a 12-month follow-up, the success rate of surgical treatment for BNC was 72%^[105]^. Redshaw *et al.* reported that among 55 patients who underwent transurethral bladder neck incision and mitomycin-C treatment, with a follow-up period of 10 months, the success rate of treating BNC was 75%^[106]^. Farrell *et al.* reported that before undergoing visual urethral incision using MMC and short-term clean intermittent catheterization, 37 patients had received at least one intervention for urethral stricture or BNC. During the median follow-up period of 23 months, 75.7% of the patients did not require additional surgical intervention^[107]^. Overall, MMC has increased the success rate of a single surgery to 72%–76%^[105–107]^, though adverse events such as tissue necrosis and allergic reactions must be considered^[35]^. While recent studies report improved success rates with intralesional antiproliferative agents (e.g., success ~ 90% with MMC injection in some series^[105]^), caution that these findings are based on small cohorts and that safety concerns temper their widespread use. Mitomycin C, an antifibrotic chemotherapeutic, has been associated with serious local complications—including cases of perivesical tissue necrosis and impaired urothelial wound healing in animal models^[108–110]^. Such effects raise concerns about using high-dose MMC in the bladder neck area. Likewise, steroid (triamcinolone) injections, which aim to reduce scarring, carry risks such as systemic absorption or rare severe reactions; indeed, there has been a report of life-threatening anaphylaxis following intralesional steroid injection^[111]^. Lacking long-term data, no standardized dosing protocols, and the fact that these treatments are typically reserved for recurrent cases due to insufficient evidence to recommend routine prophylactic use. Meta-analysis 2024 shows laser versus cold-knife offers comparable patency but ~40% less perioperative bleeding. Perform radial rather than circumferential incisions to minimize sphincter injury. Routine 14–16 Fr self-catheterization for 4–6 weeks postincision reduces early scar contraction by ~25%. Urethral stenting (e.g., UroLume®) may offer temporary patency but is associated with long-term complications including fibrosis and incontinence; thus, it is generally reserved for refractory cases^[112]^.

### Open surgical reconstruction

Open reconstructive surgery is considered the last resort for patients with complete obliteration or multiple failed endoscopic procedures^[113]^. Surgical options include retropubic or perineal approaches, often using Y–V or T-flap reconstruction or buccal mucosa grafts to widen the bladder neck^[114–116]^. Reiss *et al.*^[116]^ reported that in 10 patients with highly recurrent BNC who had undergone T-formation surgery after multiple attempts at endoscopic treatment, with a follow-up period of 26 months, the surgical success rate was as high as 100%, and the quality of life of 90% of the patients improved significantly clinically. However, open surgery is highly invasive and almost inevitably results in stress urinary incontinence(SUI), necessitating staged implantation of an artificial urinary sphincter (AUS) to restore continence^[114,117,118]^. Contemporary protocols often adopt a two-stage approach: scar excision and bladder neck reconstruction in the first stage, followed by AUS placement once tissue healing is complete. Open approaches almost invariably sacrifice the rhabdosphincter; staged AUS implantation is planned in 60%–80% to restore continence (Table 3). Although effective, this strategy significantly increases both treatment cost and surgical burden^[118,119]^. Median US in-hospital costs $28 000 versus $8500 for endoscopic repeat incision; thus reserved for tertiary centers.

**Table 3:** Efficacy and risk synopsis of endoscopic management versus open surgery for BNC

Strategy	1-yr patency	3-yr patency	Repeat intervention need	Incontinence risk
Dilation ± self-catheter	60–70%	30–40%	High	<5%
Cold knife/laser incision	65–75%	50–60% (after repeats)	Moderate	4–12%
Incision + MMC/steroid	85–90% (cumulative)	60–70%	Lower	5–10%
Open reconstruction	90–100%	85–95%	Rare	60–90%^a^
Robotic reconstruction	78–85%	70–80%^b^	Low	15–25%

^a^ Requires later AUS in majority.

^b^ Limited to follow-up ≤ 2 year in published series.

**Figure 2.**
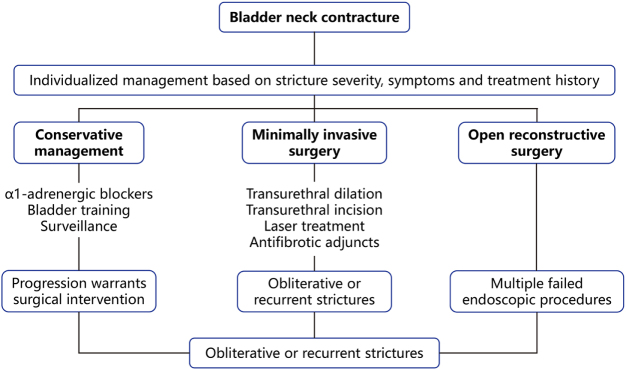
Flow chart of bladder neck contracture treatment.

In summary, BNC treatment should prioritize endoscopic and minimally invasive methods, beginning with transurethral dilation or incision combined with antifibrotic adjuncts (e.g., MMC and corticosteroids) to reduce recurrence (Table 4)^[3]^. Endoscopic approaches remain first-line owing to low morbidity, whereas open/robotic surgery provides definitive cure at the expense of higher invasiveness and continence issues. Open or robot-assisted reconstruction is reserved for obliterative or recurrent strictures unresponsive to endoscopic therapy^[2]^. Conservative strategies—including medications, bladder training, and surveillance—are appropriate only for selected patients with mild symptoms and preserved function^[120–122]^. Long-term success depends on rigorous postoperative follow-up and early intervention. All patients should undergo routine monitoring of uroflowmetry (Qmax and PVR), renal function, and symptom progression. If decreased urinary flow or voiding difficulty occurs, early outpatient dilation or incision is recommended, with cystoscopic confirmation as needed^[90]^. For patients at high risk of recurrence, intermittent self-catheterization after discharge may provide mechanical support to delay scar contraction^[123]^. Throughout the treatment continuum, strategies should be dynamically adjusted based on follow-up findings, and patients must be counseled preoperatively on potential complications and the need for staged interventions (Fig. 2). This ensures a personalized therapeutic plan that balances efficacy with quality of life^[3]^.

**Table 4:** Comparison of different treatment methods for bladder neck contracture

Therapeutic method	One-time treatment rate and recurrence	Advantages	Disadvantages
Conservative expansion (including self-expansion)	Patency rate: 55%–84%^[80,101]^ at about 1-year postinitial expansion and 3 months post-self-expansion; recurrence rate: 70%^[102]^ at about 2 years.	Simple and minimally invasive, it can temporarily relieve stenosis; suitable for initial treatment of mild stenosis^[124]^.	Patients need to adhere to long-term expansion, poor compliance, and high recurrence rate^[122]^.
Endoscopic cold knife/electrotomy	Remarkable short-term curative effect, but high recurrence rate (9.4%–16.7%)^[103]^.	Standard treatment, less trauma, can be repeated. Suitable for most moderate and severe BNC.	The recurrence rate is high, which may damage the sphincter and lead to urinary incontinence, necessitating close follow-up.
Laser incision	Single recurrence rate was documented at 2.9%^[125]^. The success rate of bladder neck incision combined with laser treatment has been reported to be up to 86%^[104]^.	Like endoscopic incision, laser bleeding is less, and cutting is fine; suitable for endoscopic treatment.	The recurrence rate is still not low, and other auxiliary means are needed to improve the curative effect.
Incision + scar inhibition (MMC or hormone)	The success rate of single surgery combined with pharmacotherapy rose to 72%–76%^[105–107]^, with the cumulative success rate of two sequential treatments being up to 85%–90%^[105,107]^.	Suitable for patients with high recurrence risk and improves the long-term effect of endoscopic treatment.	Be alert to adverse drug reactions (local necrosis, allergy, etc.).^[109–111]^
Stent placement	The initial patency rate was approximately 64.3%. Longer indwelling time (8–14 vs. 3–7 months) was significantly associated with clinical success (78.3% vs. 47.4%)^[126]^. Long-term data indicate that 10%–29% of patients require reoperation due to complications^[127–134]^.	In theory, once and for all, keep the channel open, only for a few stubborn cases.	Many complications, seldom used now.
Open surgical plasty	Small-scale studies report a 100% success rate^[116]^, with recurrence being rare during long-term follow-up.	Thorough excision of scar tissue can potentially provide a radical cure for stenosis and is suitable for cases with repeated endoscopic failure.	Trauma is significant, almost inevitably causing urinary incontinence, with an artificial sphincter often implanted in a second stage.

The success rate of treatment varies with the complexity of cases. “Success” is generally defined as postoperative urinary tract patency without further intervention. The data in the table are comprehensively reported in the literature and are not strictly controlled by the research results.

## Robotic surgery on bladder neck contracture

Robot-assisted surgery has demonstrated a profound and multidimensional impact in the management of bladder neck contracture(BNC), particularly in reducing incidence rates, optimizing anastomotic techniques, and facilitating complex reconstructions^[21]^. Robot-assisted radical prostatectomy(RARP), exemplified by the da Vinci system, has been associated with a markedly reduced incidence of BNC and vesicourethral anastomotic stricture(VUAS), owing to enhanced 3D visualization, advanced instrument articulation, and precise dissection^[135]^. Compared to open radical prostatectomy(ORP), BNC rates have declined from 5%–32% to 0%–3% following RARP^[136]^. This success is attributed to technical refinements such as continuous suturing that reduces anastomotic tension, bladder neck preservation, and minimization of intraoperative bleeding and thermal injury, all of which support optimal healing^[137,138]^.

Further technical strategies during RARP, including mucosal eversion, barbed continuous sutures for watertight anastomosis, posterior reconstruction (e.g., Rocco stitch), and maintenance of native bladder neck anatomy, contribute to the reduced risk of BNC^[21,42]^. Surgeon experience significantly influences outcomes; while complications are more common early in the learning curve, increased case volume is associated with further reductions in BNC incidence, with some centers reporting rates as low as 1.3%^[139]^.

For patients with complex or refractory BNC or VUAS, robotic platforms offer transformative reconstructive capabilities^[140]^. Transvesical RARP(TvRARP) for refractory BNC post-holmium laser enucleation has led to marked improvement in urinary flow and continence outcomes^[141]^. Single-port robotic Y-V plasty and robotic scar excision combined with free grafts or pedicled flaps (e.g., buccal mucosa) provide anatomical reconstruction with reduced recurrence and enhanced functional preservation^[142]^. Compared to open approaches, robotic reconstruction offers significant benefits including minimal blood loss, faster recovery, and better urethral sphincter preservation^[143]^. While showing promising functional outcomes (e.g., potentially lower incontinence rates compared to open surgery^[144]^), is a resource-intensive approach. Robotic surgery requires expensive equipment and specialized surgeon training, which may limit its availability to high-volume centers. The high cost and unequal access to robotic platforms mean it may not be an option for all patients or hospitals, especially in resource-limited settings.

**Figure 3.**
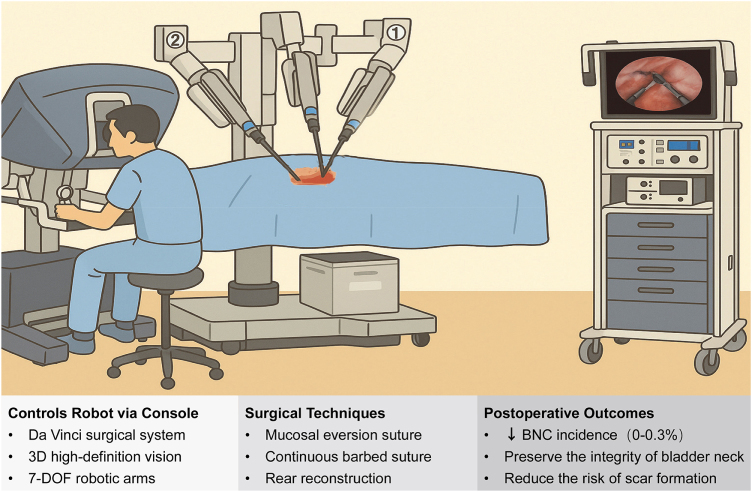
Characteristics and advantages of robotic surgery for BNC treatment.

In conclusion, robotic surgery has reshaped the landscape of BNC prevention and treatment (Fig. 3). In the context of prostate cancer, it significantly reduces stricture risk through enhanced surgical precision, and in complex or recurrent cases, it serves as a minimally invasive solution^[3]^. As technology evolves and surgical expertise accumulates, robotic platforms are expected to become the gold standard for bladder neck reconstruction and fibrotic disease management, heralding a new era of precision, safety, and individualized care^[145]^.

## Future perspectives

The future of bladder neck contracture(BNC) management is being shaped by innovations across regenerative medicine, tissue engineering, surgical technology, and targeted pharmacotherapy. Autologous adipose-derived regenerative cells (ADRCs) offer promises for modulating postoperative inflammation, inhibiting fibroblast activation, and preventing scar formation^[146]^. Liquid-phase buccal mucosa transplantation, involving suspension of mucosal cells with bioadhesives, provides a minimally invasive method of promoting urothelial repair with early promising results^[147]^.

Tissue engineering approaches, including bioabsorbable scaffolds loaded with pro-regenerative or antifibrotic molecules, aim to support structural integrity while guiding cellular behavior^[148]^. An ongoing **Phase I clinical trial** funded by the California Institute for Regenerative Medicine, which is investigating a stem cell-based, tissue-engineered urethral graft for urethral stricture disease. This trial (CLIN2-13267, https://www.cirm.ca.gov/grants/) involves seeding autologous bladder epithelial and muscle cells onto a biodegradable scaffold to create a new urethral segment. In parallel, robotic surgery continues to redefine complex reconstruction, offering precision dissection, improved graft handling, and superior functional outcomes, especially in refractory BNC/VUAS cases^[3,149,150]^.

Antifibrotic agents targeting TGF-β, LOX/LOXL, or microRNAs are emerging as adjunctive strategies that may prevent recurrence when combined with mechanical dilation or incision^[151,152]^. Personalized prevention—through patient stratification, perioperative optimization, and molecular surveillance—has the potential to reduce recurrence risk and tailor follow-up intensity. Mesenchymal stem cells have been shown to modulate fibrosis and reduce scar formation in urethral injury models—underscoring the potential of cell-based therapies to prevent contracture recurrence^[153]^. These emerging interventions remain experimental and are currently available only in research contexts. They involve sophisticated laboratory processes (cell harvesting and scaffold manufacturing) and consequently are high cost and not yet covered by insurance. Issues of regulatory approval, scalability, and long-term safety/efficacy remain to be resolved before widespread clinical adoption, while a tissue-engineered urethral graft is being tested in trials, such therapy would require significant infrastructure and expertise, which limits immediate clinical applicability.

Collectively, these strategies are converging to overcome the limitations of current therapies. Through the synergy of minimally invasive techniques, regenerative approaches, and precision interventions, the future holds the promise of more durable patency and improved quality of life for patients with BNC.

## Limitations

This review has several inherent limitations. First, its narrative design, while based on a comprehensive database search, lacks the methodological rigor of a formal meta-analysis and may be subject to selection bias. Second, the overall quality of available evidence is limited; the majority of studies on bladder neck contracture(BNC) are retrospective, single-center analyses with heterogeneous definitions of “success,” and only two prospective trials report follow-up beyond two years. Third, the exclusion of non-English publications introduces a language bias that may omit valuable regional data. Finally, while emerging therapies such as regenerative strategies and LOXL2-targeted interventions show promise, the existing evidence is limited to early-phase (≤ phase II) studies, precluding definitive conclusions regarding their efficacy and safety.

## Data Availability

The data underlying this article are available in the article.
